# Fusaric Acid immunotoxicity and MAPK activation in normal peripheral blood mononuclear cells and Thp-1 cells

**DOI:** 10.1038/s41598-017-03183-0

**Published:** 2017-06-08

**Authors:** Shanel Dhani, Savania Nagiah, Dhaneshree B. Naidoo, Anil A. Chuturgoon

**Affiliations:** Discipline of Medical Biochemistry, School of Laboratory of Medicine and Medical Sciences, College of Health Science, University of KwaZulu-Natal, Durban, South Africa

## Abstract

Fusaric acid (FA), a food-borne mycotoxin, is a potent divalent metal chelator. The human immune system is complex and susceptible to environmental insult however, the immunotoxity of FA remains unknown. We investigated the immunotoxicity of FA on human peripheral blood mononuclear cells (PBMCs) and Thp-1 cells. FA was cytotoxic to PBMCs (IC_50_-240.8 μg/ml) and Thp-1 (IC_50_-107.7 μg/ml) cells at 24 h. FA induced early apoptosis but significantly decreased caspase activity in PBMCs, a characteristic of paraptosis. In Thp-1 cells, FA induced apoptosis and increased caspase −9 and −3/7 activities. In PBMCs, FA maintained mitochondrial membrane potential and decreased protein expression of Bax whilst increasing expression of p-Bcl-2; FA induced oxidative stress and depleted ATP levels in both cell types. In Thp-1 cells, FA increased mitochondrial membrane depolarization and decreased p-Bcl-2 expression. In PBMCs, FA significantly up-regulated the MAPK protein expression of p-ERK and p-JNK but down-regulated p-p38 expression. In Thp-1 cells, FA up-regulated MAPK protein expression of p-ERK whilst p-JNK and p-p38 expression were down-regulated. In conclusion FA induced programmed cell death and altered MAPK signaling in healthy PBMCs and Thp-1 cells strongly suggesting a possible mechanism of FA induced immunotoxicity *in vitro*.

## Introduction

Endophytic fungi produce mycotoxins that are toxic to animals and humans^1^. Fusaric acid (FA, 5-butylpicolinic acid), is a picolinic acid derivative produced by several strains of *Fusarium* species^2, 3^. These fungal strains are ubiquitous in soil and are known to parasitize maize and many other cereal grains^4, 5^.

FA contains a pyridine ring with a butyl side chain that allows it to easily permeate cell membranes^6^. The toxicity of FA is also due to its ability to chelate divalent ions such as magnesium, calcium, zinc and iron^2, 7^. The nitrogen in the pyridine ring and the deprotonated, negatively charged oxygen on the carboxylic acid group are responsible for FA’s divalent metal chelating ability^8, 9^.

The human immune system functions in host defense against environmental exposure to bacteria, viruses, parasites, fungi and other perturbations, and in acquiring immunity against invading pathogens^10, 11^. In response to foreign particle or pathogen, several signaling pathways are activated in immune cells^12^. Foremost of these pathways, is the activation of mitogen-activated protein kinases (MAPKs)^12^. MAPK activity directs diverse immune responses ranging from stress, cell death/survival and immune defense^12–14^.

Optimal cellular mitochondrial function increases ATP synthesis and reactive oxygen species (ROS) that mediate cell signaling pathways^8^. The amount of intracellular ROS will significantly influence the MAPK pathway^6^. The MAPK family comprises of three universal serine/threonine protein kinases; these include the extracellular signal-regulated kinase (ERK), c-Jun N-terminal kinase (JNK) and p38 kinase^15, 16^; each group of MAPK is activated via a series of phosphorylation events^16^. The first event involves the phosphorylation and activation of a MAPK kinase kinase (MAPKKK), which in turn, phosphorylates and activates a MAPK kinase (MAPKK). MAPKKs activate MAPKs through dual phosphorylation on both threonine and tyrosine residues located within the tri-peptide motif of the MAPK^14, 15, 17, 18^. Once activated, MAPKs phosphorylate several transcription factors, thereby regulating gene expression and cellular functions^13, 14^.

Apoptosis is executed by immune cells to maintain homeostasis of the immune system^19–21^. Apoptosis occurs via two main pathways, the intrinsic and extrinsic apoptotic pathways^19, 22, 23^. Both the intrinsic and extrinsic pathways are activated by caspases; the initiator caspases (−8 and −9) are involved in the intrinsic pathway, whilst the executioner caspases (−3/7) are integral to the extrinsic pathway^19, 24^. Paraptosis is distinct from necrotic and apoptotic cell death and its features are defined by the lack of apoptotic morphology and independent of caspase activation^19, 22, 23, 25–27^.

The phytotoxicity of FA is well documented and includes altered mitochondrial membrane potential and inhibition of ATP synthesis^28, 29^. In animals, FA inhibits the activity of dopamine-β-hydroxylase, synthesis of nucleic acids (zinc finger proteins involved in DNA repair) and impairs protein synthesis^30^. In young swine, FA showed moderate toxicity, induced vomiting and increased concentration levels of tryptophan and serotonin in the brain^31^. Elevated levels of serotonin results from its impaired regulation and consequently amplifies behaviors distinctive of the firing of serotonergic neurons such as loss of appetite and lethargy^32^. In zebrafish, FA induced teratogenic effects by inhibition of lysyl oxidase (a copper-dependent enzyme)^33^. FA also decreased norepinephrine levels in the brain, heart, spleen and adrenal gland of rats^34^.

To date, no study has investigated the effect of FA on the mammalian immune system. In this study, we assessed the immunotoxicity of FA associated with MAPK activity in healthy human peripheral blood mononuclear cells (PBMCs) and the acute monocytic leukemic (Thp-1) cell line. It was hypothesized that FA altered MAPK signaling was immunotoxic in both cell types. This study shows that FA, a common food borne mycotoxin, is toxic to the human immune system. This data may help develop a better understanding of the immune risks associated with FA consumption. This is of importance in South Africa, the epicenter of infectious diseases, where the majority population relies on maize as a food staple.

## Results

### Cell viability of PBMCs and Thp-1 cells

**Figure 1:**
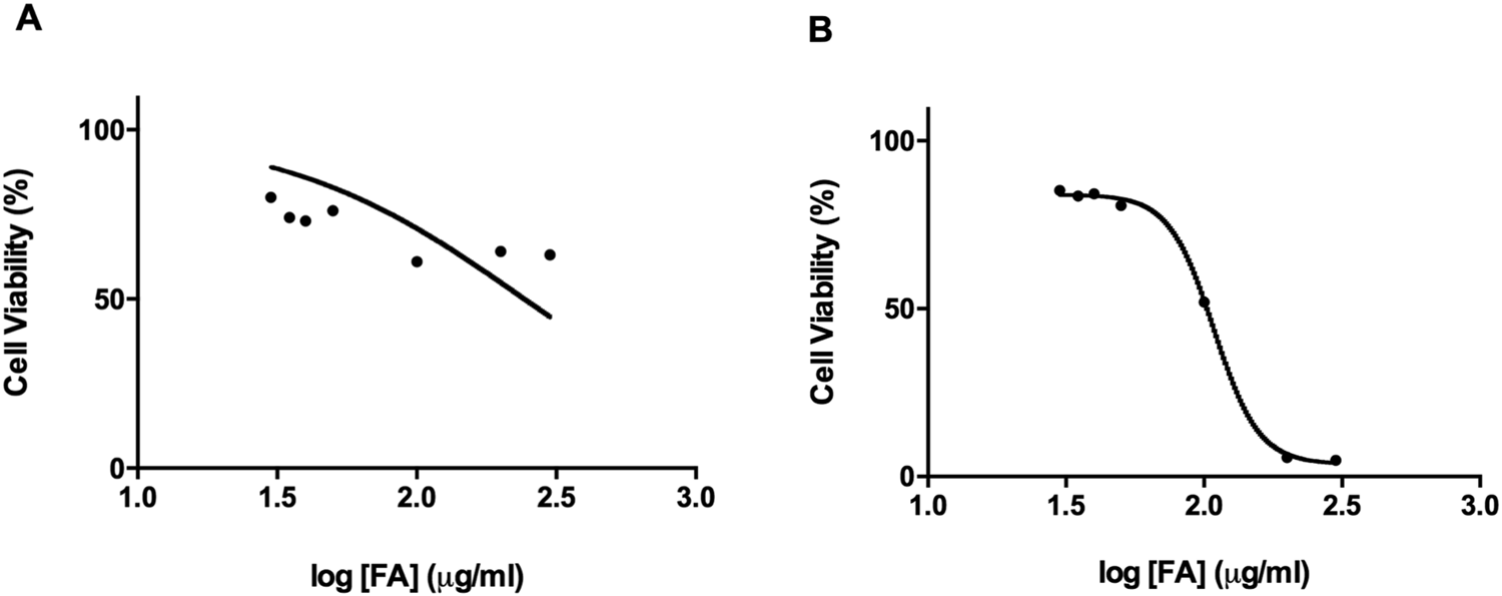
Cytotoxicity of FA on PBMCs and Thp-1 cells. FA induced a dose dependent decrease in PBMC (**A**) and Thp-1 (**B**) cell viability.

The WST-1 assay showed that FA induced a dose dependent decrease in PBMC and Thp-1 cell viability over 24 h (Supplementary Tables: S3-PBMC, S4-Thp-1). Thp-1 cells were more susceptible than PBMCs to FA toxicity. An IC_50_ of 240.8 μg/ml (Fig. 1A) and 107.7 μg/ml (Fig. 1B) determined for PBMCs and Thp-1 cells respectively; and was used in all subsequent assays.

### FA activates caspase-independent cell death in PBMCs and intrinsic apoptosis in Thp-1 cells

**Figure 2:**
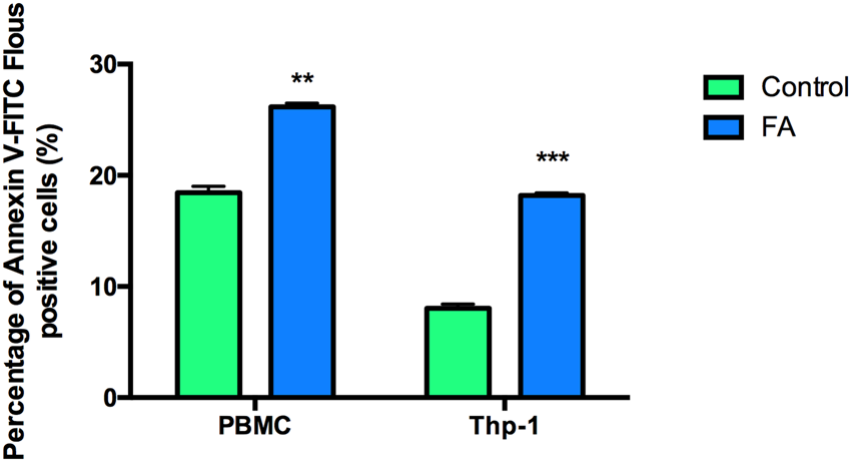
Translocation of PS in PBMCs and Thp-1 cells. FA induced PS externalization in both PBMCs and Thp-1 cells. Data are expressed as mean ± SD. **p < 0.005 relative to respective control; ***p < 0.0001 relative to respective control.

To confirm the toxicity of FA, we assessed the externalization of phosphatidylserine (PS) on the plasma membranes of PBMCs and Thp-1 cells. FA significantly increased the externalization of PS in PBMCs and Thp-1 cells by 1.42 (18.43 + 0.006% vs. 26.16 + 0.003%; *p* = 0.0003) and 2.27 (8.03 + 0.004% vs.18.19 + 0.002%; *p* < 0.0001) fold, respectively (Fig. 2). Propidium iodide (PI) staining showed a decreased percentage of necrotic cells by FA as compared to the controls in both PBMCs and Thp-1 cells (Supplementary Fig. S1). This was confirmed by quantifying the release of lactate dehydrogenase (LDH). FA did not induce membrane leakage in both PBMCs and Thp-1 cells (Supplementary Fig. S2), therefore, necrotic cell death was excluded.

Next, we measured caspase −8, −9 and −3/7 activities to determine the type of programmed cell death induced by FA in PBMCs and Thp-1 cells. Interestingly, despite the increase in PS externalization, FA significantly decreased caspase −8 activity by 0.81 fold (1.09 ± 0.001 × 10^4^ RLU vs. 0.88 ± 0.042 × 10^4^ RLU; *p* = 0.0022), caspase −9 by 0.73 fold (10.89 ± 0.609 × 10^4^ RLU vs. 7.92 ± 0.241 × 10^4^ RLU; *p* = 0.0070) and caspase −3/7 activities by 0.10 fold (1.19 ± 0.258 × 10^4^ RLU vs. 0.12 ± 0.026 × 10^4^ RLU; *p* = 0.0035) in PBMCs, relative to the control (Table 1). This result suggests that FA induced caspase-independent cell death in PBMCs. In Thp-1 cells, however, FA significantly decreased caspase −8 activity by 0.74 fold (4.52 ± 0.306 × 10^4^ RLU vs. 3.3265 ± 0.021 × 10^4^ RLU; *p* = 0.0211), but significantly increased both caspase −9 activity (1.43 fold; 62.67 ± 3.701 × 10^4^ RLU vs. 89.37 ± 0.590 × 10^4^ RLU; *p* = 0.0065) and caspase −3/7 activity (5.33 fold; 0.82 ± 0.482 × 10^4^ RLU vs. 4.38 ± 0.604 × 10^4^ RLU; *p* = 0.0041) when compared to the control (Table 2); an indicator of activation of intrinsic apoptosis.

**Table 1:** Effect of FA on caspase (−8, −9, −3/7) activity in healthy PBMCs.

	Mean ± SD (RLU × 10^4^)		Fold change	*p* value
	PBMC			
	Control	FA		
Caspase −8	1.0918 ± 0.0007	0.8831 ± 0.0419	0.81	0.0022^**^
Caspase −9	10.8855 ± 0.6094	7.9185 ± 0.2409	0.73	0.0070^*^
Caspase −3/7	1.1858 ± 0.2581	0.1218 ± 0.0261	0.10	0.0035^**^

SD: standard deviation; RLU: relative light units; *p < 0.05; **p < 0.005.

**Table 2:** Effect of FA on caspase (−8, −9, −3/7) activity in Thp-1 cells.

	Mean ± SD (RLU × 10^4^)		Fold change	*p* value
	Thp-1			
	Control	FA		
Caspase −8	4.5235 ± 0.3055	3.3265 ± 0.0206	0.74	0.0211^*^
Caspase −9	62,6683 ± 3.7013	89.3652 ± 0.5900	1.43	0.0065^*^
Caspase −3/7	0.8210 ± 0.4816	4.3758 ± 0.6041	5.33	0.0041^**^

SD: standard deviation; RLU: relative light units; *p < 0.05; **p < 0.005.

### FA induces oxidative stress in PBMCs and Thp-1 cells

**Figure 3:**
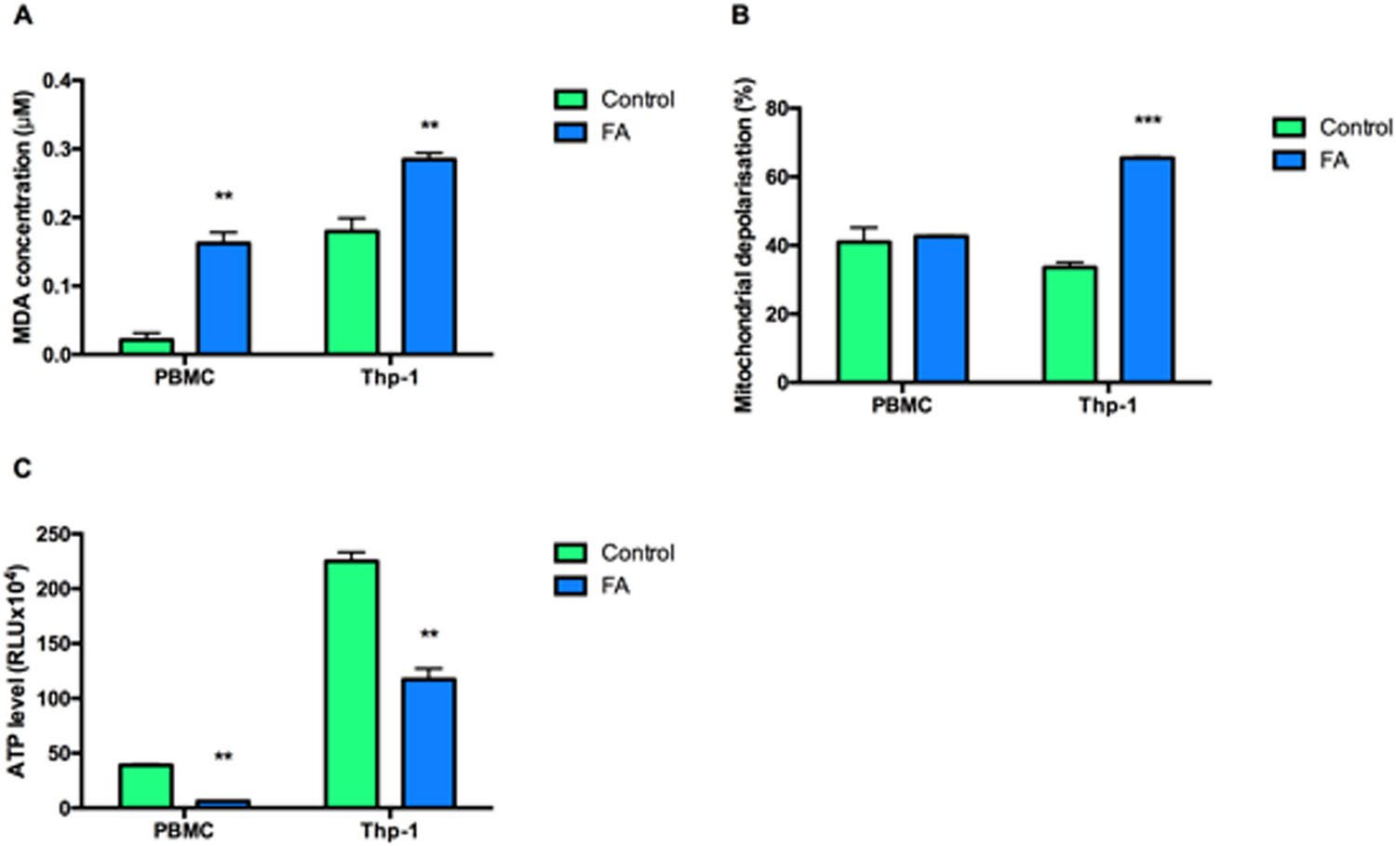
Effect of FA on the oxidative status and mitochondrial function in PBMCs and Thp-1 cells. FA increased MDA levels in both PBMCs and Thp-1 cells (**A**) had no effect on the mitochondrial membrane potential in PBMCs but increased depolarization of the mitochondrial membrane in Thp-1 cells (**B**). FA depleted ATP levels in PBMCs and Thp-1 cells (**C**). Data are expressed as mean ± SD. **p < 0.005 relative to respective control; ***p < 0.0001 relative to respective control.

Increased intracellular ROS is known to promote oxidative stress, that is not only injurious to cells but may also regulate cell signaling pathways^8^. Lipid peroxidation, a marker for oxidative stress, was measured by quantifying malondialdehyde (MDA) using the Thiobarbituric acid (TBARS) assay. FA significantly elevated MDA levels in PBMCs (7.59 fold; 0.02 ± 0.010 μM vs. 0.16 ± 0.016 μM; *p* = 0.0006) and Thp-1 cells (1.59 fold; 0.18 ± 0.020 μM vs. 0.28 ± 0.010 μM; *p* = 0.0039) (Fig. 3A). FA induced oxidative stress in both normal human PBMCs and Thp-1 cells.

Mitochondria are important in maintaining cellular redox homeostasis and activation of the intrinsic apoptotic pathway. The evaluation of FA induced mitochondrial membrane integrity by flow cytometry in PBMCs showed that despite increased MDA levels, there was no effect on the mitochondrial membrane potential; however, in Thp-1 cells, mitochondrial membrane depolarization was significantly increased (1.95 fold; 33.58 ± 1.425% vs. 65.48 ± 0.329%; *p* = 0.0007) when compared to the controls (Fig. 3B).

Further, FA significantly depleted ATP levels in both PBMCs (0.16 fold; 38.97 ± 1.183 × 10^4^ RLU vs. 6.11 ± 0.266 × 10^4^ RLU; *p* = 0.0002) and in Thp-1 cells (0.52 fold; 225.21 ± 8.014 × 10^4^ RLU vs. 117.26 ± 10.017 × 10^4^ RLU; *p* = 0.0007) relative to controls (Fig. 3C).

### Effect of FA on Bax and p-Bcl-2 protein expression in PBMCs and Thp-1 cells

**Figure 4:**
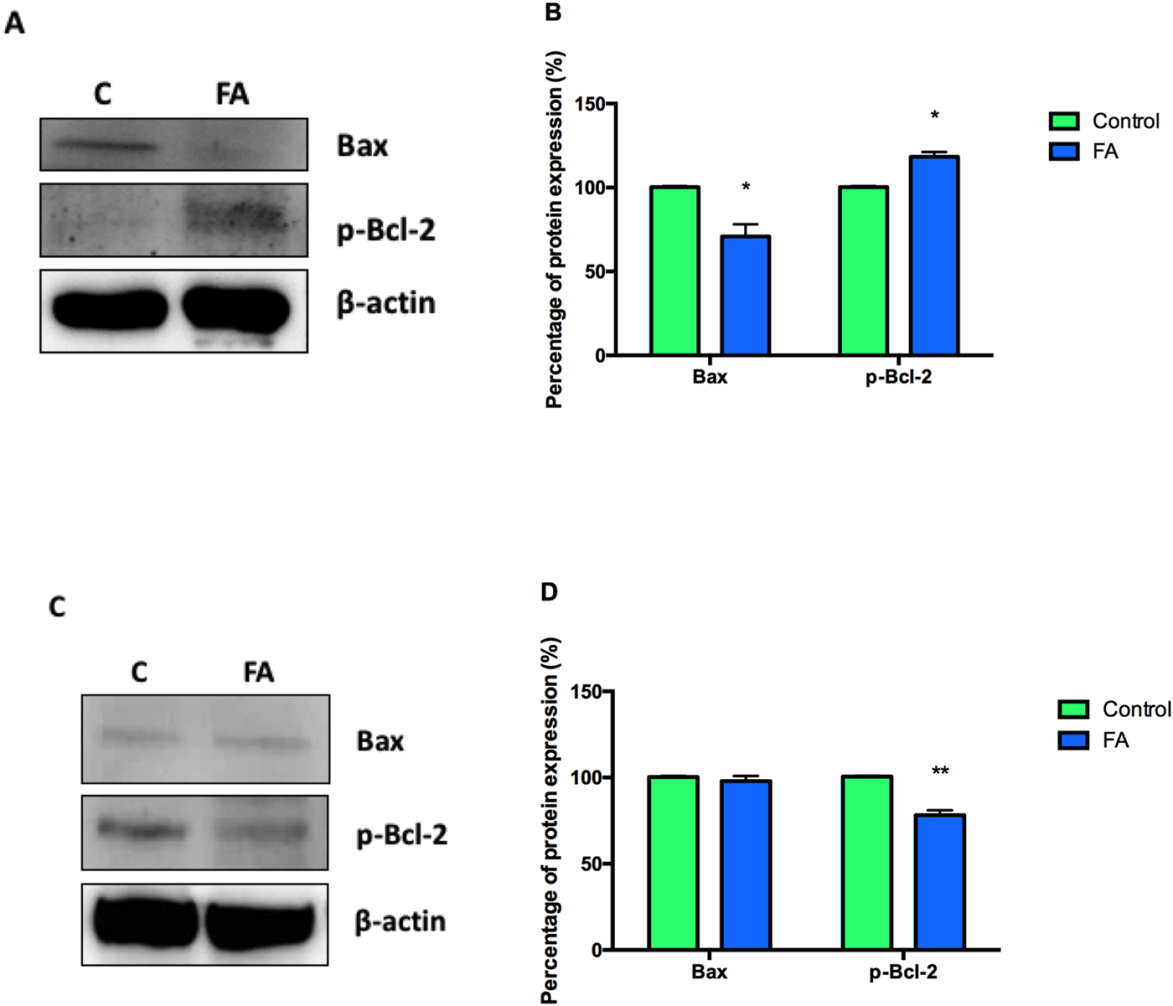
Effect of FA on apoptotic regulator proteins Bax and p-Bcl-2 in PBMCs and Thp-1 cells. Protein expressions of Bax and p-Bcl-2 in healthy PBMCs (**A**) and Thp-1 cells (**C**). Percentage of Bax and p-Bcl-2 protein expression in healthy PBMCs (**B**) and Thp-1 cells (**D**). Percentage of protein expressions were represented as mean ± SD. *p < 0.05 relative to respective control; **p < 0.005 relative to respective control.

To validate caspase-dependent and –independent cell death, protein expressions of pro-apoptotic Bax and anti-apoptotic p-Bcl-2 was determined. FA significantly decreased Bax protein expression (0.71 fold; 100 ± 10.33% vs. 70.90 ± 7.34%; *p* = 0.0201) and increased p-Bcl-2 protein expression (1.18 fold; 100 ± 6.40% vs. 118.30 ± 2.83%; *p* = 0.0455) in PBMCs compared to the control (Fig. 4A and B), whilst it only significantly decreased the protein expression of p-Bcl-2 (0.78 fold; 99.39 ± 0.89% vs. 77.40 ± 2.82%; *p* = 0.0007) in Thp-1 cells (Fig. 4C and D).

### Effect of FA on MAPKs expression in PBMCs and Thp-1 cells

**Figure 5:**
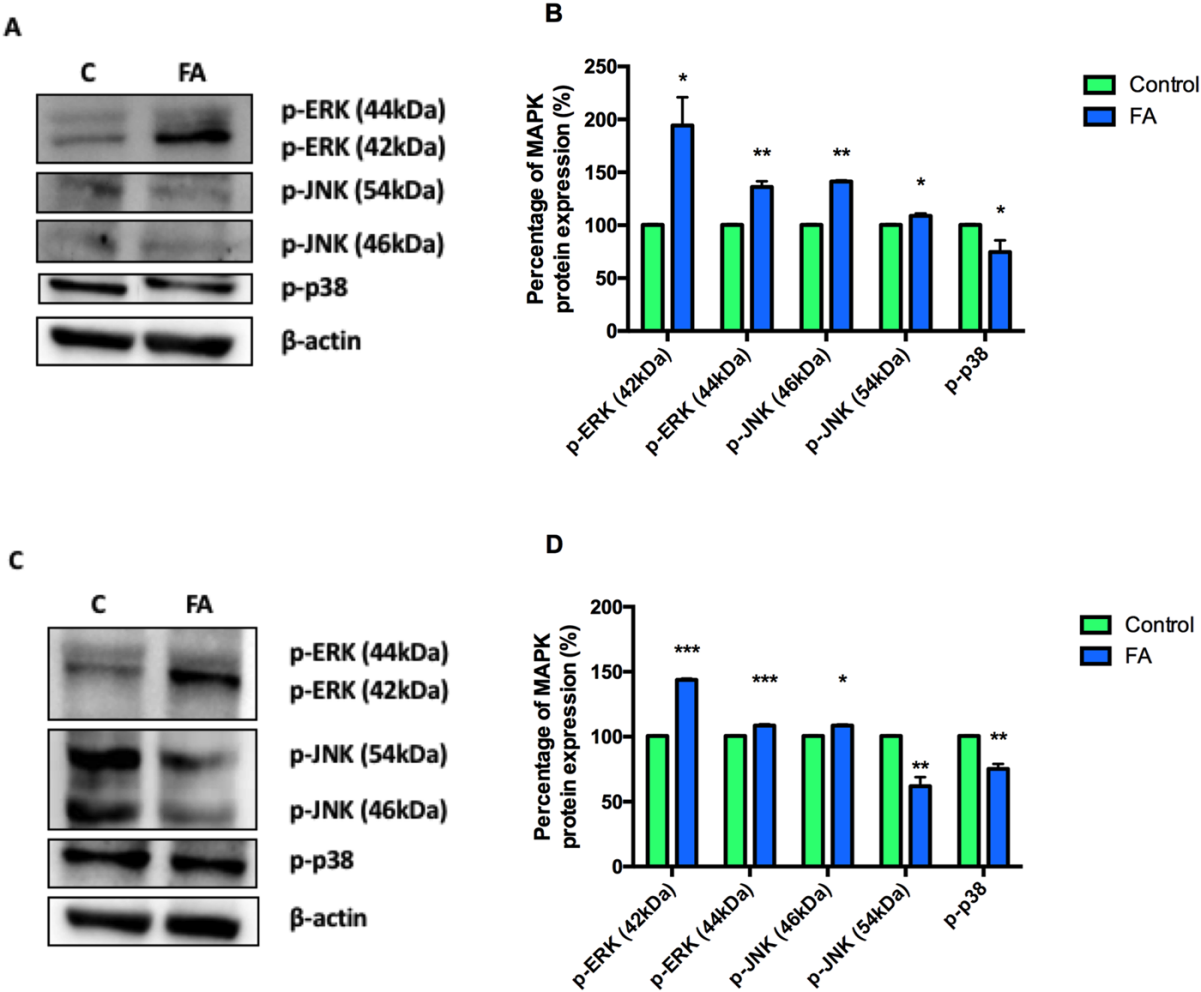
Effect of FA on MAPK protein expression. MAPK protein expression in healthy PBMCs (**A**) and Thp-1 cells (**C**). Percentage of MAPK protein expression in healthy PBMCs (**B**) and Thp-1 cells (**D**). Percentage of protein expressions were represented as mean ± SD. *p < 0.05 relative to respective control; **p < 0.005 relative to respective control; ***p < 0.0001 relative to respective control.

Finally, to evaluate the effect of FA on MAPK signaling, phosphorylation of MAPK protein expressions was determined using western blotting. In PBMCs, FA significantly increased expression of p-ERK (42 kDa fragment and 44 kDa fragment; 1.94 fold; 100 ± 5.73% vs. 194.19 ± 26.83%; *p* = 0.0271 and 1.36 fold; 99.80 ± 0.68% vs. 136.20 ± 5.29%; *p* = 0.0006, respectively) and p-JNK (46 kDa fragment and 54 kDa fragment; 1.46 fold; 97.10 ± 10.06% vs. 141.39 ± 0.92%; *p* = 0.0035 and 1.13 fold; 96.56 ± 11.92% vs. 108.77 ± 2.45%; *p* = 0.0454, respectively) (Fig. 5A and B). In Thp-1 cells, FA also significantly increased p-ERK (42 kDa and 44 kDa fragments; 1.35 fold; 100 ± 3.54% vs. 135.13 ± 15.06%; *p* < 0.0001 and 1.05 fold; 100 ± 5.15% vs. 104.68 ± 6.93%; *p* = 0.0006, respectively) (Fig. 5C and D), whilst significantly decreasing p-JNK (46 kDa and 54 kDa fragments; 1.02 fold; 105.85 ± 20.25% vs. 108.37 ± 0.75%; *p* = 0.0461 and 0.62 fold; 103.48 ± 12.05% vs. 63.98 ± 7.03%; *p* = 0.0055, respectively) (Fig. 5C and D). Furthermore, FA significantly decreased the expression of p-p38 in PBMCs and Thp-1 cells (0.75 fold; 100 ± 9.17% vs. 74.73 ± 11.08%; *p* = 0.0401 and 0.70 fold; 100 ± 17.40% vs. 69.86 ± 9.89%; *p* = 0.0033, respectively) (Fig. 5A–D).

## Discussion

The promising role of divalent ion chelators in proliferative and virulent diseases has led to growing interest^35–37^. FA, a picolinic acid analogue and potent divalent metal chelator, has shown potential as an anti-cancer, anti-microbial and anti-viral agent^35–37^. Recently, the role of divalent ion chelators in proliferative and virulent diseases has been extensively studied. However, the toxicity of FA, a food-borne mycotoxin, on the immune system containing a diverse population of cells, has to date not been studied.

In this study, we showed the immunotoxic potential of FA to both healthy PBMCs (diverse population of immune cells) and the distinct immune Thp-1 cells. Interestingly, FA inhibited Thp-1 cell proliferation at an IC_50_ value less than half that of PBMCs (Fig. 1A and B). This result is in agreement with other studies that showed cytotoxic effects of FA on WI-38 cells (fibroblastic cells), LoVo cells (colorectal adenocarcinoma cells) and MDA-468 cells (human breast adenocarcinoma cells) in which FA had preferentially inhibited the proliferation of cancerous cells (LoVo and MDA-468) when compared to the normal cells (WI-38)^35^. Our data suggests that FA may exert selective toxicity to distinct immune cell types as evidenced by the Thp-1 response, albeit a leukemic cell line. Additionally, in comparison to the anti-neoplastic drug, ellipticine failed to inhibit PBMC and Thp-1 cell viability; which further exemplifies the potency of FA.

Contrary to the study by Fernandez-Pol (1998), FA significantly increased the externalization of PS in both PBMCs and Thp-1 cells by 26.16% and 18.19%, respectively (Fig. 2). Given that the externalization of PS occurs during both apoptosis and paraptosis, activities of caspases −8, −9 and −3/7 were assayed to determine type of cell death induced by FA in both cell lines; apoptosis requires caspase activation whilst paraptosis is independent of caspase activation. FA substantially decreased caspase activities in PBMCs (Table 1), strongly suggesting that paraptosis was the preferred mode of cell death. In Thp-1 cells, however, FA significantly increased caspase −9 and −3/7 activities were (Table 2), indicating the induction of intrinsic apoptosis in Thp-1 cells.

Immune cells respond to stimuli by activating MAPK signaling to amplify other signals to elicit an appropriate physiological response for programmed cell death^14, 38^. Previous studies showed that prolonged activation of MAPK signaling induced cell death via ROS-activation of MAPK signaling pathways^16^. ROS are continuously generated by cellular processes, with the mitochondrion being the major source^6^. Excessive ROS generated during oxidative phosphorylation can cause oxidative damage to proteins, DNA and phospholipids^16^; oxidative degradation of lipids results in the formation of lipid peroxides such as MDA^39^. FA significantly increased MDA levels in both PBMCs and Thp-1 cells (Fig. 3A), indicative of a prolonged oxidative stress environment. Furthermore, FA disrupted mitochondrial membrane potential by increasing mitochondrial depolarization in Thp-1 cells (Fig. 3B). This could be due to the weak acidic nature of the carboxylic acid group of FA^40^. Weak acids act as proton carriers across lipid membranes, thereby disrupting the proton gradient along the electron transport chain (ETC)^41, 42^. Interestingly, FA did not disrupt the mitochondrial membrane potential in normal PBMCs (Fig. 3B) despite the significant depletion of ATP levels in both normal PBMCs and Thp-1 cells (Fig. 3C). In Thp-1 cells, this is substantiated by the decreased cell viability (decreased redox potential) and increased mitochondrial membrane depolarization. Also, the activation of ATP dependent caspases −9 and −3/7 may further deplete ATP levels^43^. In PBMCs, however, the decreased ATP levels may be due to increased activation and prolonged activation of ATP dependent protein kinases.

Intracellular ROS not only alters cellular integrity but is also important to MAPK signaling cascades^6^; FA induced increased ROS production and up-regulated protein expression of ERK in Thp-1 cells (Fig. 5C and D). Although ERK signaling pathways are well known for their role in promoting cell survival, recent studies have demonstrated their ability to potentiate apoptosis^44^. Prolonged activation of ERK may be due to the inhibition of tyrosine phosphatases, a group of enzymes responsible for the removal of phosphate groups on phosphorylated tyrosine residues, hence inactivating the protein^3^. However, tyrosine phosphatases are sensitive to increased ROS and become oxidized, thereby inhibiting their activity and prolonging ERK activation^3, 45^.

JNK and p38 MAPK signaling pathways are generally directed towards initiating cell death upon activation by stress signals. Recently, however, these signaling pathways have been associated in both cell death and survival^38^. In Thp-1 cells, FA significantly decreased p-JNK activation and p-p38 protein expressions (Fig. 5C and D). A study by Pedram *et al*. (1998) documented the cross-talk between the ERK and JNK MAPKs where the activation of JNK by ERK MAPK was followed by the activation of ERK by vascular endothelial growth factor (VEGF) whilst JNK stimulated ERKs proliferative signaling. Therefore, it can be inferred that a decrease in JNK activity hinders the cross-talk between JNK and ERK MAPKs, preventing survival signaling by ERK^46^.

Additionally, JNK and ERK MAPKs regulate the expression of Bcl-2 family proteins that are central in regulating the mitochondrial apoptotic death pathway^3, 47^. Bcl-2 inhibits apoptosis by forming a complex with pro-apoptotic proteins such as Bax^47, 48^. Phosphorylation of Bcl-2 compromises it’s protein stability and affects dimerization with Bax^49^. Thus, dissociation from the complex at the mitochondrial membrane leads to the formation of mitochondrial permeability transition pore (mPTP) and subsequent caspase activation^50^. In support of the increased caspase −9 and −3/7 activities in Thp-1 cells, FA decreased p-Bcl-2 expression resulting in apoptotic cell death (Fig. 4C and D). JNK signaling regulates the expression of Bcl-2 and is up-regulated in response to JNK activation. FA activated ERK death signaling, decreased p-Bcl-2 expression and induced apoptosis in Thp-1 cells, but had no significant effect on Bax expression (Fig. 4C and D). This may be due to the deletion of the p53 gene in the Thp-1 cell line as p53 acts as a transcription factor for Bax expression and recruitment to the mitochondrial membrane^24, 51–55^. Additionally, increased ERK activity regulates mitochondrial membrane potential^56^ and corresponds with the increased caspase −9 and −3/7 activities, and the subsequent activation of cell death in Thp-1 cells by FA. In PBMCs, FA increased p-Bcl-2 expression (Fig. 4A and B) and decreased Bax expression (Fig. 4A and B), with a corresponding decrease in caspase −8, −9 and −3/7 activities. Increased p-Bcl-2 expression helps maintain the mitochondrial membrane integrity and subsequent mitochondrial membrane potential by preventing the release of cytochrome c, activation of caspase −9 and the initiation of intrinsic apoptosis, further validating the induction of paraptosis in normal PBMCs by FA.

Although the molecular activation of paraptosis remains unknown, studies have suggested the involvement of MAPK signaling in the induction of cell death. In PBMCs, FA significantly increased the expression of ERK and JNK whilst decreasing p38 expression (Fig. 5A and B). Sperandio *et al*., (2000) reported that ERK and JNK activity mediated paraptosis stimulation by insulin-like growth factor 1 receptor, and that inhibition of these MAPKs prevented the induction of paraptosis in 293 T cells^57^. Another study by Yumnam *et al*., (2014) showed the involvement of ERK MAPK in hesperidin-induced paraptosis of human hepatocellular carcinoma (HepG2) cells^58^. Sugimori and colleagues (2015) recently showed that activated JNK induced paraptosis induction in HL-60 and NB4 human promyelocytic leukemic cell lines and in bone marrow blasts treated with benfotiamine^59^. Contrary to the studies by Sperandio *et al*., (2000) and Yumnam *et al*., (2014), benfotiamine inhibited the activity of ERK in bone marrow blasts and had no effect on ERK activity in HL-60 and NB4 cell lines^57, 58^. This suggests that the involvement of MAPK in the induction of paraptosis may be dependent on the cell line and type of activation. Additionally, caspase −9 was reported to be a direct target of ERK MAPK, and that phosphorylation at threonine 125 on caspase −9 inhibits its pro-apoptotic activity^60^. These findings support the activation of MAPK signaling pathways in the induction of paraptosis in PBMCs treated by FA.

## Conclusion

FA is immunotoxic to both healthy PBMCs and Thp-1 cells, albeit at a higher concentration in PBMCs. The cancerous Thp-1 cells are highly susceptible to FA toxicity. Collectively, the results show that the host response to FA exposure augmented MAPK signaling and induction of apoptosis in Thp-1 cells (via the mitochondrial apoptotic pathway) and paraptosis in PBMCs. This study shows that FA, a common food borne mycotoxin, is toxic to the human immune system. This data may help develop a better understanding of the immune risks associated with FA consumption. This has great importance in socio-economically challenged countries where the majority population relies on corn as a food staple.

## Material and Methods

### Materials

Cell culture reagents for PBMC maintenance and FA (*Gibberella fujikuroi*) were purchased from Sigma Aldrich (Johannesburg, SA). The Thp-1 cells and media were purchased from ATCC (University Boulevard Manassas, USA) and Scientific group (Johannesburg, SA), respectively. Luminometry reagents were obtained from Promega (Madison, USA).

### Cell culture

PBMCs were isolated from whole blood using Histopaque 1077 (Sigma Aldrich) and gradient centrifugation from young healthy males following institutional (University of KwaZulu-Natal) ethical approval (BE057/15) and written informed consent. Collection and use of blood was in compliance with relevant institutional guidelines and procedures. Isolated PBMCs were maintained at 37 °C with 5% CO_2_ in RPMI 1640 medium (supplemented with 10% FCS, 1% L-glutamine and 1% pencillin-streptomycin). Thp-1 cells were cultured in RPMI 1640 medium supplemented with 10% FCS, 1% L-glutamine, 1% pencillin-streptomycin, 1 mM sodium pyruvate and 0.05 mM β-mecaptoethanol. Thp-1 cells were maintained at 3 × 10^5^ cells/ml in 75 cm^3^ ventilated flasks at (37 °C, 5% CO_2_) and were split at a cell count of 8 × 10^5^ cells/ml. Viability of cells was assessed using trypan blue exclusion.

### Cell viability

The cytotoxicity of FA on PBMCs and Thp-1 cells was analyzed using the WST-1 reagent. Briefly, PBMC and Thp-1 cells (20,000 cells/well) were seeded into a 96-well microtitre plate. The cells were incubated with varying FA concentrations (30–300 µg/ml) in triplicate (200 µl/well) for 24 h (37 °C, 5% CO_2_). Ellipticine, anti-neoplastic agent, was used as a positive control to FA toxicity (Data shown in Supplementary Tables: S1 PBMC; S2 Thp-1). A positive control of cells with RPMI only and a negative control with RPMI/WST-1 reagent solution was also seeded. Following incubation, the plate was centrifuged at 24 °C, 400*xg* for 10 min. The supernatant was then aspirated and 110 µl/well of a RPMI/WST-1 reagent solution (1:10) was added and incubated for 3 h (37 °C, 5% CO_2_). The optical density of the colorimetric reaction was measured at a wavelength of 450 nm and reference wavelength of 620 nm using a spectrophotometer (Bio-Tek uQuant, Winooski, VT, USA). The percentage cell viability was calculated by standardizing untreated (control) cells to 100% and then comparing FA treated cells to the control cells (Detailed calculation shown as Supplementary Information). The concentration of half maximum inhibition (IC_50_) was determined using GraphPad Prism v5.0 software. All assays were performed in triplicate, twice independently.

### Cell death parameters

#### PS Externalization

Flow cytometry was performed to determine the externalization of PS. Following treatment, 100 μl of an Annexin V-FITC Fluos solution (1:1:50; annexin V-FITC: PI: staining buffer) was added to each sample (200,000 cells in 100 μl PBS) and incubated in the dark at room temperature (RT, 15 min). Thereafter, the samples (20,000 events) were analyzed for apoptosis on the AccuriTM C6 flow cytometer. The cells were gated to exclude cellular debris using the Fl-1 channel (525 nm) (BD Biosciences, Johannesburg, SA). The results were expressed as a percentage.

#### LDH activity

The LDH cytotoxicity detection kit (Roche, Mannheim, Germany) was used to confirm damaged/necrotic cells. In brief, cell homogenates (100 μl) were seeded into a 96-well opaque polystyrene microtitre plate in triplicate. Subsequently, 100 μl of a substrate mixture containing a catalyst (diaphorase/NAD^+^) and dye solution (INT/sodium lactate) was added to each homogenate and incubated in the dark for 25 min (RT). The optical density was measured (500 nm) using a spectrophotometer (Bio-Tek uQuant, Winooski, VT, USA). The results were reported as mean optical density.

#### Caspase activity

Caspase activities of −8, −9 and −3/7 were determined using luminometry. Cells (20,000 cells/well) were seeded into a 96-well opaque polystyrene microtitre plate in triplicate. 20 µl/well of the reagent (Caspase-Glo® 3/7, Caspase-Glo® 8 and Caspase-Glo® 9 Assays) was added to each sample and incubated in the dark for 30 min (RT). Thereafter, the luminescence was measured on a Modulus™ microplate luminometer (Turner Biosystems, Sunnyvale, USA). All data was expressed as relative light units (RLU).

### Evaluation of oxidative stress

#### Lipid peroxidation

The TBARS was used to determine FA generation of ROS^61^. Briefly, cell homogenates (400 μl) were added to a 7% phosphoric acid (400 μl) and a thiobarbituric acid (1%W w/v)/butylated hydroxytoluene (0.1 mM) (TBA/BHT) solution. A positive control containing MDA (1 μl) and a negative control containing 3 mM hydrogen chloride were prepared. All samples were heated in a water bath (100 °C, 15 min) and allowed to cool (RT). Thereafter, lipids were extracted with butanol (1.5 ml) and were measured on a spectrophotometer (Bio-Tek uQuant, Winooski, VT, USA) at 532 nm with reference wavelength of 600 nm. The mean optical density for each sample was calculated and divided by the absorption coefficient (156 mM^−1^). The results were expressed in μM.

#### Mitochondrial membrane potential

Mitochondrial membrane potential was measured using the JC-1 Mitoscreen kit (BD Biosciences, Johannesburg, SA) and flow cytometry. Briefly, 100 µl of a JC-1 working solution was added to each sample (200,000 cells in 100 µl PBS) and incubated in the dark for 30 min (RT). Following incubation, 100 µl flow cytometry sheath fluid was added to each sample and were analyzed on the AccuriTM C6 flow cytometer. A total of 20 000 events were gated using AccuriTM C6 flow cytometer Fl-1 channel (525 nm) (BD Biosciences, Johannesburg, SA). The results were expressed as a percentage.

#### ATP levels

Intracellular ATP levels were measured using the ATP CellTitre Glo reagent (Promega, Madison, USA). Following treatment, 20,000 cells/well were seeded into a 96-well opaque polystyrene microtitre plate in triplicate. The reagent (20 μl/well) was added to each sample and incubated in the dark for 30 min (RT). Thereafter, the luminescence was measured on a ModulusTM microplate luminometer (Turner Biosystems, Sunnyvale, USA). The data was expressed as relative light units (RLU).

### Western blotting

Western blots were performed to analyze the protein expressions of p-ERK, p-JNK, p-p38, Bax and p-Bcl-2. Briefly, total protein was isolated using Cytobuster^TM^ reagent (Novagen, San Diego, CA, USA). Cells were re-suspended in 200 µl Cytobuster and incubated on ice for 30 min. Following incubation, the cells were centrifuged for 10 min (180xg, 4 °C). Protein samples were quantified by the bicinchoninic acid (BCA) assay and standardized to 0.2 mg/ml (PBMCs) and 1.0 mg/ml (Thp-1). The samples were then boiled in Laemmli Sample buffer (dH_2_0, 0.5 M Tris-HCl (pH 6.8), glycerol, 10% SDS, β-mercaptoethanol, 1% bromophenol blue) for 5 min (100 °C). Thereafter, the samples (25 μl- Thp-1; 40 μl- PBMC) were electrophoresed in sodium dodecyl sulphate polyacrylamide (SDS-PAGE) gels (4% stacking gel, 7.5% resolving gel) for 1.5 h at 150 V (Bio-Rad compact power supply). The separated proteins were electro-transferred onto nitrocellulose membranes for 30 min (400 mA) using the Trans-Blot Turbo Transfer system (Bio-Rad). The membranes were then blocked for 1 h (RT) with 5% bovine serum albumin (BSA) in Tris Buffer Saline with tween 20 (TTBS- NaCl, KCl, Tris, dH_2_O, 0.5% tween 20, pH 7.5) or 5% Non-Fat Dry Milk (NFDM) in TTBS for phospho- and non-phospho- antibodies, respectively. Thereafter, the membranes were incubated with primary antibody [mouse anti-p-ERK (9106), mouse anti-p-JNK (9255), rabbit anti-Bax (5023), rabbit anti-p-Bcl-2 (2827), Cell Signalling, 1: 1000; mouse anti-p-p38 (M8177), β-actin (A3854), Sigma Aldrich, 1: 5000] for 1 h (RT) and then overnight at 4 °C. The membranes were washed 5 times with TTBS (10 min intervals) and incubated (RT) with horseradish peroxidase (HRP)- conjugated secondary antibody [goat anti-rabbit (ab6112), anti-mouse (ab97046), Abcam, 1: 5000] for 1 h. Once more, the membranes were washed 5 times with TTBS (10 min intervals). Protein band images were detected using Clarity Western ECL Substrate (Bio-Rad) and captured using Alliance 2.7 Image documentation system (UViTech, Cambridge, UK). The expression of protein bands was analyzed using UViBand Advanced Image Analysis software v12.14 (UViTech, Cambridge, UK). All proteins were normalized to β-actin before comparison (i.e. control vs. FA treatment. The data was expressed as relative band intensity (RBI).

### Statistical analysis

Statistical analysis was performed using GraphPad Prism v5.0 software (GraphPad Software Inc., La Jolla, USA). GraphPad Prism Software was used for the unpaired t-test with Welch’s correction to assess the differences between samples. Level of significance (*p*) was established at a *p* < 0.05.

## Electronic supplementary material

Supplementary dataset
