# Supplementary material for: Fusaric Acid immunotoxicity and MAPK activation in normal peripheral blood mononuclear cells and Thp-1 cells
Source: Sci Rep. 2017 Jun 8;7:3051. doi: 10.1038/s41598-017-03183-0 (PMC5465181; doi:10.1038/s41598-017-03183-0)
Supplement: Supplementary file 1 — Supplementary dataset [file 41598_2017_3183_MOESM1_ESM.doc]

**Fusaric Acid immunotoxicity and MAPK activation in normal peripheral blood mononuclear cells and Thp-1 cells**

Shanel Dhani^1^, Savania Nagiah^1^, Dhaneshree B. Naidoo^1^, Anil A. Chuturgoon^1*^

^1^Discipline of Medical Biochemistry, School of Laboratory of Medicine and Medical Sciences, College of Health Science, University of KwaZulu-Natal, South Africa

**Supplementary information**

**Detailed calculation of percentage cell viability**

The percentage cell viability was calculated as follows:

% cell viability =
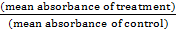
 x 100

**Supplementary tables**

**Table S1: Cell viability of PBMCs treated with ellipticine for 24 hours.**

| **Ellipticine concentration (µg/ml)** | **Average optical density (OD) + Standard deviation (SD)** | **Cell viability (%)** |
| --- | --- | --- |
| **0** | 0,290 **+** 0,016 | - |
| **Vehicle control (0,1% DMSO)** | 0,309 **+** 0,004 | 100 |
| **0,01** | 0,301 **+** 0,032 | 97,41 |
| **0,025** | 0,315 **+** 0,034 | 102,05 |
| **0,05** | 0,314 **+** 0,015 | 101,62 |
| **0,1** | 0,319 **+** 0,003 | 103,13 |
| **0,25** | 0,299 **+** 0,015 | 96,87 |
| **0,6** | 0,322 **+** 0,000 | 104,21 |
| **0,8** | 0,310 **+** 0,008 | 100,22 |

**Table S2: Cell viability of Thp-1 cells treated with ellipticine for 24 hours.**

| **Ellipticine concentration (µg/ml)** | **Average optical density (OD) + Standard deviation (SD)** | **Cell viability (%)** |
| --- | --- | --- |
| **0** | 0,258 **+** 0,019 | - |
| **Vehicle control (0,1% DMSO)** | 0,271 **+** 0,012 | 100 |
| **0,01** | 0,272 **+** 0,024 | 100,62 |
| **0,025** | 0,265 **+** 0,010 | 97,78 |
| **0,05** | 0,269 **+** 0,006 | 99,51 |
| **0,1** | 0,290 **+** 0,030 | 107,27 |
| **0,25** | 0,269 **+** 0,011 | 99,26 |
| **0,6** | 0,250 **+** 0,004 | 92,49 |
| **0,8** | 0,274 **+** 0,010 | 101,23 |

**Table S3: Cell viability of PBMCs treated with FA for 24 hours.**

| **FA concentration (µg/ml)** | **Log [FA] concentration** | **Average optical density (OD) + Standard deviation (SD)** | **Cell viability (%)** |
| --- | --- | --- | --- |
| **0** |  | 0,340 **+** 0,048 | 100 |
| **30** | 1,477121255 | 0,274 **+** 0,014 | 80,41 |
| **35** | 1,544068044 | 0,252 **+** 0,037 | 74,05 |
| **40** | 1,602059991 | 0,250 **+** 0,036 | 73,36 |
| **50** | 1,698970004 | 0,262 **+** 0,013 | 76,89 |
| **10** | 2 | 0,210 **+** 0,003 | 61,80 |
| **200** | 2,301029996 | 0,221 **+** 0,009 | 64,94 |
| **300** | 2,477121255 | 0,218 **+** 0,004 | 63,96 |

**Table S4: Cell viability of Thp-1 cells treated with FA for 24 hours.**

| **FA concentration (µg/ml)** | **Log [FA] concentration** | **Average optical density (OD)** | **Cell viability (%)** |
| --- | --- | --- | --- |
| **0** |  | 2,492 **+** 0,105 | 100 |
| **30** | 1,477121255 | 2,1223 **+** 0,117 | 85,19 |
| **35** | 1,544068044 | 2,082 **+** 0,192 | 83,57 |
| **40** | 1,602059991 | 2,099 **+** 0,059 | 84,23 |
| **50** | 1,698970004 | 2,010 **+** 0,205 | 80,68 |
| **10** | 2 | 1,294 **+** 0,165 | 51,93 |
| **200** | 2,301029996 | 0,140 **+** 0,010 | 5,63 |
| **300** | 2,477121255 | 0,120 **+** 0,006 | 4,83 |

**Supplementary figures**


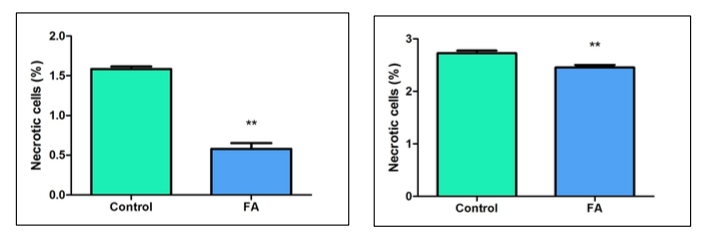


**Figure S1:** **Effect of FA on necrotic cell death in PBMCs (left) and Thp-1 cells (right). ***p*<0,005.**


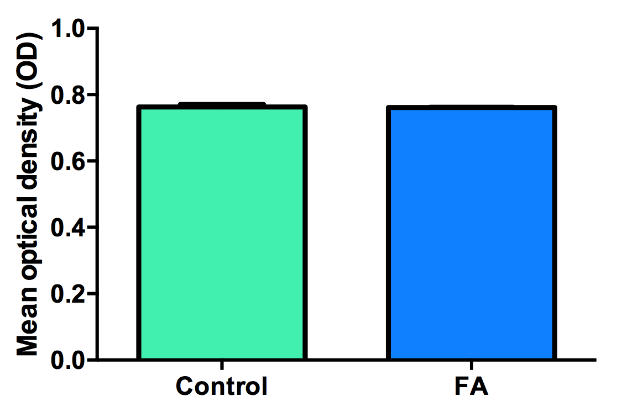

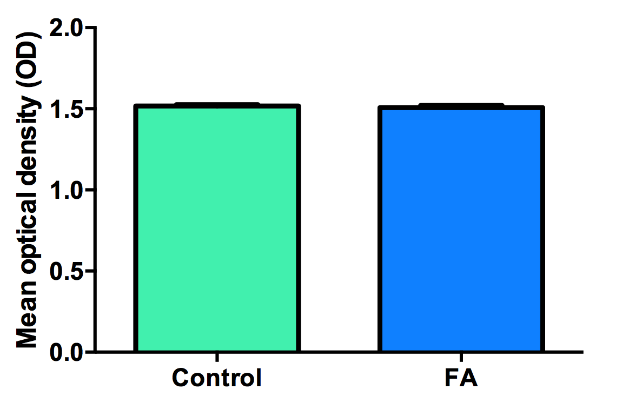


**Figure S2: Quantification of LDH in PBMCs (left) and Thp-1 cells (right) treated with FA for 24 hours.**
